# Peritoneal dialysis-associated peritonitis caused by *Mycobacteroides massiliense*: the first case and review of the literature

**DOI:** 10.1186/s12882-021-02297-y

**Published:** 2021-03-12

**Authors:** Shintaro Hamada, Tomoaki Takata, Tsuyoshi Kitaura, Chiori Teraoka, Akio Aono, Sosuke Taniguchi, Yukari Mae, Hajime Isomoto, Hiroki Chikumi, Satoshi Mitarai

**Affiliations:** 1 Division of Gastroenterology and Nephrology, Tottori University Faculty of Medicine, 36-1 Nishimachi, Yonago, Tottori, 683-8504 Japan; 2 Division of Infectious Diseases, Tottori University Faculty of Medicine, Yonago, Tottori, 683-8504 Japan; 3 Department of Clinical Laboratory, Tottori University Hospital, Yonago, Tottori, 683-8504 Japan; 4 The Research Institute of Tuberculosis, Japan Anti-tuberculosis Association, Kiyose, Tokyo, 204-0023 Japan

**Keywords:** Peritonitis, *Massiliense*, *Abscessus*, Nontuberculous Mycobacteroides

## Abstract

**Background:**

Peritoneal dialysis (PD)-associated peritonitis caused by nontuberculous Mycobacterium is rare; however, the number of cases has increased over the past decades. *Mycobacteroides massiliense* is a subspecies of the *Mycobacteroides abscessus* complex. It has different clinical characteristics compared to the other subspecies of the complex. Previous case reports of PD-associated peritonitis caused by *Mycobacteroides abscessus* complex have not distinguished the subspecies in detail.

**Case presentation:**

A 40-year-old man presented with an exit-site and tunnel infection refractory to antibiotic therapy. Peritonitis occurred after simultaneous catheter removal and reinsertion. The *Mycobacteroides abscessus* complex was detected in the culture of the dialysis effluent. Removal of the PD catheter combined with antibiotics, including macrolides, resulted in a good clinical course. Further analysis of multiplex PCR and the hsp65 gene sequence identified the bacterium as *Mycobacteroides massiliense*.

**Conclusions:**

The *Mycobacteroides abscessus* complex is classified into three subspecies; *Mycobacteroides abscessus*, *Mycobacteroides massiliense*, and *Mycobacteroides bolletii*. These have different characteristics, particularly antibiotic susceptibility. Therefore, clear identification of the subspecies of *the Mycobacteroides abscessus* complex is necessary for definitive treatment.

## Background

Peritoneal dialysis (PD)-associated peritonitis is a major complication leading to PD failure. The most common organisms causing peritonitis are *S. aureus*, *Enterococcus*, *Escherichia. coli*, and *Pseudomonas aeruginosa*. Relatively fewer cases caused by nontuberculous mycobacterium (NTM) have been reported [1, 2]. A *mycobacteroides abscessus* complex is a group of NTM that can cause peritonitis related to poor prognosis than the other NTM [2]. Therefore, appropriate treatment is necessary for peritonitis caused by bacteria. The *M. abscessus* complex has three subspecies: *Mycobacteroides abscessus*, *Mycobacteroides massiliense*, and *Mycobacteroides bolletii* [3]. Since the three subspecies have different susceptibilities to antibiotics, an increasing number of investigations have emphasized the importance of their definite identification. In this report, we present the first case of *M. massiliense* peritonitis with a review of previous case reports describing peritonitis caused by *M. abscessus* complex.

## Case presentation

**Fig. 1:**
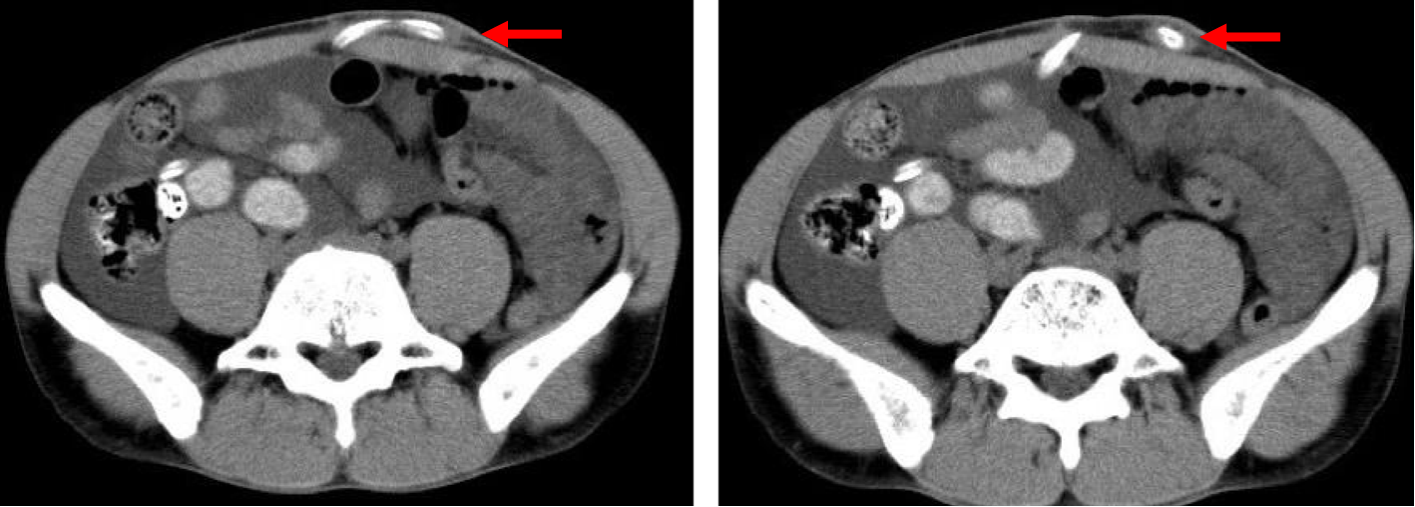
Images of computed tomography. Increased density observed around the peritoneal dialysis catheter (arrows)

**Fig. 2:**
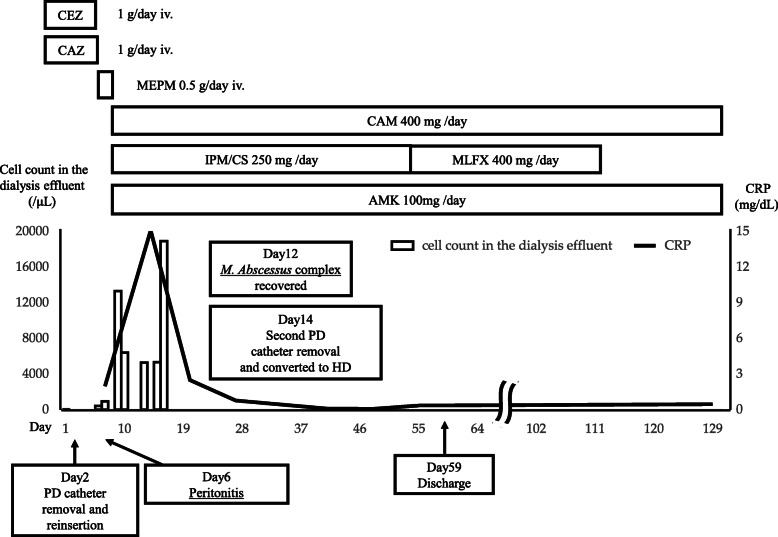
The patient’s clinical course. CEZ: cefazolin; CAZ: ceftazidime; MEPM: meropenem; CAM: clarithromycin; IPM/CS: imipenem/cilastatin; MFLX: moxifloxacin; AMK: amikacin; CRP: C-reactive protein; HD: hemodialysis; PD: peritoneal dialysis

A 40-year-old male patient with PD was admitted to our hospital because of a persisting exit-site and tunnel infection of the PD catheter. He was started on PD due to IgA nephropathy 8 months before admission. He had no comorbid disorders and was not receiving corticosteroids. He visited the outpatient unit because of the pain at the exit-site of the PD catheter 1 month before admission. He was administered levofloxacin (250 mg once every other day) orally for 7 days, followed by oral cefpodoxime proxetil (100 mg once every other day) for 8 days and oral minocycline (100 mg twice daily) for 8 days, combined with topical nadifloxacin for 1 month. However, his symptoms worsened. On admission, his blood pressure was 130/90 mmHg, pulse was 79 beats/min and body temperature was 36.6 °C. The skin at the exit-site of the PD catheter and the subcutaneous cuff was red, painful, swollen, and purulent discharge from the exit-site was observed, indicating exit-site infection and catheter-tunnel infection. He did not have rebound tenderness. Laboratory data from the whole blood showed a white blood cell count of 4900/μL with 84% segmented neutrophils and a C-reactive protein level of 0.11 mg/dL. The dialysis effluent was clear, and the cell count of the dialysate effluent was 16/μL. Therefore, peritonitis was not suspected. An increased density around the PD catheter was observed on abdominal computed tomography (Fig. 1). Cultures of pus and dialysis effluent were negative. On the second day, the patient underwent simultaneous PD catheter removal and reinsertion. Gram staining of the removed catheter showed negative results. On day 6, abdominal pain and a fever of 38.3 °C appeared. The dialysis effluent became turbid, and the cell count of the dialysate increased to 379/μL with 40% neutrophils; thus, PD-associated peritonitis was strongly suspected. Empirical therapy with intravenous cefazolin (1 g once daily) and ceftazidime (1 g once daily) was initiated, although the antibiotics were switched to intravenous meropenem (0.5 g once daily) because of exacerbation of the abdominal pain and an increase in the cell count of the effluent to 13,272/μL. On day 10, *Cutibacterium acnes* was cultured from the subcutaneous cuff, deep cuff and infected tissue around the exit-site. In addition, acid-fast bacilli were detected in the same specimens. The culture from dialysis effluent collected on day 6 was positive for *M. abscessus* by the Bruker MALDI Biotyper (Bruker Daltonics, Billerica, MA) matrix-assisted laser desorption ionization-time of flight mass spectrometry (MALDI-TOF MS) system. On day 12, all three cultures, including the dialysis effluent collected on day 6, subcutaneous cuff and deep cuff removed on day 2, turned out to be positive for *M. abscessus*. Therefore, meropenem was changed to oral clarithromycin (200 mg twice daily), intravenous imipenem/cilastatin (250 mg once daily), and intravenous amikacin (100 mg once daily). After the removal of the PD catheter on day 14, his symptoms rapidly improved. Renal replacement therapy was switched to hemodialysis (HD), and the dosage of amikacin was changed to 300 mg after each HD session. On day 55, imipenem/cilastatin was changed to oral moxifloxacin (400 mg once daily) due to mild hepatic dysfunction; aspartate aminotransferase, 63 IU/L; alanine aminotransferase, 20 IU/L; and lactate dehydrogenase, 300 IU/L. The patient was discharged from the hospital on day 59. Combined therapy with clarithromycin, amikacin, and moxifloxacin was continued until day 114, and moxifloxacin was discontinued according to the susceptibility of the bacterium (Table 1) [4]. We further analyzed the bacterium by multiplex PCR and the hsp65 gene sequence and identified it as *M. massiliense* [5]. Clarithromycin and amikacin were continued 17 weeks. The patient does not plan to re-start PD concerning for recurrence of peritonitis and encapsulation peritoneal sclerosis, and is receiving maintenance hemodialysis without recurrence of peritonitis (Fig. 2).

**Table 1:** Susceptibility to antibiotics

Antibiotics	MIC (μg/mL)	Clinical categorization
Sitafloxacin	8	–
Moxifloxacin	> 8	R
Cefmetazole	64	–
Amikacin	4	S
Clarithromycin	0.06	S
Linezolid	32	R
Imipenem	32	R
Doxycycline	> 16	R
Minocycline	> 8	–

*MIC* Minimal inhibitory concentration, *S* Susceptible, *R* Resistant

## Discussion and conclusions

Herein, we report the first case of PD-associated peritonitis caused by *M. massiliense*, a subspecies of the *M. abscessus* complex. The susceptibility to antibiotics and pathogenicity varies in each individual. Therefore, it is necessary to identify these subspecies and pay attention to their susceptibility especially macrolides.

*The M. abscessus* complex belongs to the Runyon classification group IV, which rapidly grows within 7 days [6]. The *M. abscessus* complex has been isolated from surface water, tap water, and soil [7]. The major clinical manifestations of *M. abscessus* complex are skin and soft-tissue infections and respiratory infections, and only a few cases of peritonitis have been documented [8]. Rapidly growing NTM comprises approximately 3% of the causative pathogens of PD-associated infections [9]. The *M. abscessus* complex accounts for 8.8% of NTM-caused peritonitis and is associated with poor outcomes [2]. Considering the number of NTM peritonitis cases is increasing [10, 11], it is important to appropriately manage peritonitis caused by *M. abscessus* complex.

*The M. abscessus* complex is resistant to several antibiotics. In addition to surgical removal of the infected foci, it is recommended to treat patients with multiple agents including macrolides [8]. Yoshimura et al. reported that 82.1% of peritonitis or exit-site infections require catheter removal [12]. Furthermore, a case of exit-site infection requiring catheter removal after the termination of antibiotic therapy has been reported [13]. Considering that almost all the peritonitis cases failed to continue PD and that some cases resulted in patient death [9, 12], it seems necessary to remove the PD catheter when the *M. abscessus* complex is isolated.

Recently, *M. abscessus* complex has been classified into three subspecies; *M. abscessus, M. massiliense,* and *M. bolletii* [3, 14]. These subspecies cannot be distinguished by MALDI-TOF MS, which is usually used for clinical isolates; therefore, previous reports of *M. abscessus* complex are a mixture of three subspecies. Multiplex PCR targeting several primer sets enabled clear identification of the M. abscessus complex [5]. Clinical behavior, particularly susceptibility to antibiotics, differs among each subspecies [15]. *M. massiliense* responded well to macrolides, whereas *M. abscessus* was resistant [16]. Similarly, *M. abscessus* lead to poor outcomes due to resistance to clarithromycin, whereas *M. massiliense* was susceptible [15]. *M. abscessus* possesses a gene responsible for inducible resistance to macrolides [17]; thus, treatment with macrolides must be carefully determined when treating *M. abscessus* complex. Furthermore, *M. massiliense* has been reported to cause outbreaks [18]. Therefore, a clear identification of *M. massiliense* is necessary.

In the present case, the patient showed exit-site and trans-catheter infections without any signs of peritonitis. Peritonitis appeared after simultaneous removal and reinsertion of the catheter. The patient was not immunocompromised. The long duration of oral antibiotics before admission may have influenced the emergence of NTM. As the culture of the pus and dialysate effluent was negative on admission, it is important to suspect acid-fast bacilli when the culture-negative infection persists. In the present case, *M. abscessus* was initially recovered from the culture; however, it was later identified as *M. massiliense*. To the best of our knowledge, this is the first case report of PD-associated peritonitis caused by *M. massiliense.* Further consideration of this organism would lead to better treatment of peritonitis in the future.

## Data Availability

All data generated or analysed during this study are included in this published article.

## Abbreviations

CRP: C-reactive protein
HD: Hemodialysis
MALDI-TOF MS: Matrix-assisted laser desorption ionization-time of flight mass spectrometry
NTM: Nontuberculous mycobacterium
PD: Peritoneal dialysis
